# Conversion of asymptomatic infection to symptomatic visceral leishmaniasis: A study of possible immunological markers

**DOI:** 10.1371/journal.pntd.0008272

**Published:** 2020-06-18

**Authors:** Vidya Nand Rabi Das, Sanjiva Bimal, Niyamat Ali Siddiqui, Ashish Kumar, Krishna Pandey, Sanjay Kumar Sinha, Roshan Kamal Topno, Vijay Mahentesh, Ashish Kumar Singh, Chandra Shekhar Lal, Subhankar Kumar Singh, Pradeep Das

**Affiliations:** Rajendra Memorial Research Institute of Medical Science (ICMR), Patna, India; Centro de Pesquisa Gonçalo Moniz-FIOCRUZ/BA, BRAZIL

## Abstract

**Introduction:**

Presence of asymptomatic individuals in endemic areas is common. The possible biomarkers in asymptomatic individuals once they get exposed to infection as well as following conversion to symptomatic disease are yet to be identified.We identified asymptomatic Visceral leishmaniasis (VL) infection amongst rK39^+^sorted direct agglutination test positive (DAT^+^) endemic healthy population and confirmed it by quantitative PCR(qPCR).The immunological determinants such as Adenosine deaminase (ADA), Interferon gamma (IFN-γ), Tumour Necrosis Factor alpha (TNF-α) and Interleukin 10 (IL-10)were examined to predict probable biomarkers for conversion to symptomatic VL.

**Methods:**

Sample size was 5794 healthy individuals from VL endemic region. Antibody tests(DAT &rK39) were performed and later a qPCR assay was employed using kDNA specific primers and probes. Immunological biomarkers examined were ADA level by ADA–MTP kit and quantitative cytokines(IFN-γ, IL-10 and TNF-α) by ELISA.

**Results:**

120 asymptomatic individuals of 308 rK39 sero-positives were DAT positive comprising of 56 with previous history and 64 with no history of VL. RT-PCR confirmed asymptomatic VL in 42 sero-positives. These were followed up through repeated qPCR and evaluation of immunological determinants. We observed10 symptomatic cases converted from a total of 42 asymptomatic individuals identified at base-line. The level of ADA, IL-10 and IFN-γ remained consistently high in asymptomatic cases and amongst these, ADA and IL-10 but not IFN-γ remained higher at the development of clinical symptoms into active VL. On the contrary, there was no significant change in the mean concentration of TNF-α at both stages of the disease.

**Discussion:**

We surmise from our data that considerable proportion of asymptomatic cases can be a reservoir and may play a crucial role in transmission of visceral leishmaniasis in endemic areas.

The data also suggests that ADA and IL-10 can serve as a potential biomarker during the conversion of asymptomatic into symptomatic VL.

## Author summary

The most threatening form of leishmaniasis is human visceral leishmaniasis, which is caused by *L*. *donovani* in Indian subcontinent. The disease accounts for huge annual burden of infectious disease in India. Efforts towards disease elimination programme are far beyond satisfaction and there is need to re-strengthen health monitoring, surveillance programme in endemic areas. There is a greater need to identify asymptomatic individuals amongst endemic healthy control by active surveillance programme. Further, except a few, many asymptomatic subjects become sero-negative without developing VL due to strong inherent cellular immunity possessed by them. This study enumerates use of rK39, DAT and quantitative PCR(qPCR) in asymptomatic detection and then evaluate immunological biomarkers(Adenosine deaminase, IFN-γ, TNF-α and IL-10) which help in disease conversion to symptomatic VL, at 6 month follow- up. Follow–up analysis revealed that antibody testing with rK39 (3.24%), DAT (8.33%) had poor agreement with disease conversion. By qPCR, we could identify the level of parasitemia, and collectively with serology, the test detected 23.80% of asymptomatic cases converted into disease. The level of ADA and IL-10 remained consistently high during conversion. IFN-γ became high in asymptomatic infection but became low on conversion, whereas, no significant change in level of TNF-α was observed at both stages of disease. Thus by use of qPCR, concomitantly with DAT over rK39^+^ sorted endemic healthy control may be employed for early case detection. Adenosine is produced by ecto-nucleotide during *L*.*donovani*infection, has been previously reported and in parallel to this study we conclude that determination of adenosine deaminase level (ADA) might help identify early cases with more tendency to convert into disease. This will help to boost health monitoring programme to eradicate Kala-azar in Bihar, India.

## Introduction

Leishmaniasis is a disease caused by protozoan parasite of genus *Leishmania* caused by 20 *Leishmania* species which are transmitted to humans by the bites of infected *Phlebotomus* sand flies. In Indian sub-continent, Kala-azar or human visceral leishmaniasis (VL) caused by *L*. *donovani* is most fatal if left untreated. In India, the disease is compounded further by toxicity, drug resistance, relapse and appearance of post kala-azar dermal leishmaniasis (PKDL) in 5–15 years after treatment [1,2]. A major proportion of many exposed persons, harbour the parasite but remain the asymptomatic leaving only a small number of individuals to exhibit clinical manifestation [3, 4]. Therefore asymptomatic cases are wide spread in VL endemic areas [5,6]. Asymptomatic cases were also earlier reported to harbour the parasite in their blood [7].

In the absence of animal reservoir, asymptomatic VL and PKDL patients in particular, are thought to be reservoir for visceral leishmaniasis in Indian sub-continent and this necessitates an accurate diagnosis of such cases prevalent in endemic areas [8, 9]. Previously, the direct demonstration of parasite was based on microscopy or culture of blood and aspiration(bone marrow,lymph-nodes or spleen). The sensitivity of such tests varies considerably and is associated with risks of serious bleeding especially the splenic aspiration and should only be carried out at settings with surgical services. Although many specific diagnostic tests for confirmed VL patients exist, but these tests do not address the time and proportion of asymptomatic individuals who will be clinically manifest[3, 5]. This necessitates comprehensive longitudinal studies to identify the components which will dictate the conversion from asymptomatic to symptomatic VL. The diagnosis of VL has been further advanced by molecular tools such as PCR [10] and qPCR (<10% to up to 30%) [11, 12]. More importantly, the most of asymptomatic individuals are identified from areas, where VL remains endemic and even exceed the number of symptomatic cases [13–18]. Such cases assume importance as probable reservoir of VL transmission in endemic areas but on account of ethical issues and invasiveness, these tests cannot be identify the time of conversion of these subjects into symptomatic and the associated components which can influence conversion[3,5]. Many immunological methods such as direct agglutination test (DAT) and lateral flow immunochromatographic tests, such as rK39 and rkE-16 have been introduced to screen large number of individuals in endemic areas [19–23]. Commercially available rapid diagnostic tests are sensitive and specific with acceptable reproducibility and most are heat stable (WHO/ TDR, 2011). Such serological tests accompanied with a PCR assay can be well applied in identification of asymptomatic individuals living in endemic areas.

The issue, how only a few exposed asymptomatic individuals but not the all develop full blown disease manifestations is still unexplored. One study showed the identical pattern of immune response of asymptomatic cases and cured VL but there were certain changes in the level of ADA in asymptomatic individuals versus cured VL [24, 25].

The Main thrust of the study was to identify the key immunological Biomarkers for the emergence of asymptomatic cases and the factors that lead to their conversion into clinically manifestation of the disease. The assay of ADA activity in the serum and other biological fluids is reported to be useful in many pathological conditions [26, 27]. Cell mediated immunity (CMI) plays an important role, where causative organism grows intra-cellularly for instance, VL caused by *L*. *donovani* and reports also have suggested importance of ADA as a potential marker of cellular immunity.

Mononuclear cells are known to secrete ADA in, response to *Leishmania* infection[28] and our previous reports have also shown that marginal numbers of asymptomatic individuals develop cell mediated immunity and the elevated serum activity for ADA is associated in conversion of asymptomatic cases into symptomatic VL [1]. Immuno-pathogenesis of VL is determined by cytokines produced during cellular immune response to identify the subsequent resistance or susceptibility of a VL patient to disease [29]. The inability of a VL patient to mount protective Th^1^ immune response (IL-2 IFN-γ, TNF- α), exacerbates the pathogenicity and in some cases results in resistance to drug [1, 30].There are reports that when over expression of IL-10, render the resistant mice, susceptible to infection [31]. Both the IL-10 and IL-6 response is triggered which upregulates in all forms of leishmaniasis and their level subsides as the patients recover from the disease [32,33].

Attempt has been made in the present study to elucidate the key immunological factors that promote the conversion of asymptomatic individuals to symptomatic stage of visceral leishmaniasis. We demonstrate that a seizable proportion of asymptomatic cases can be reservoir and may play a crucial role in transmission of visceral leishmaniasis in endemic areas.

## Materials and methods

### Study site and sample size

Two endemic areas in Bihar state: Kothia and Pirari, Saran, two known villages with high endemicity for VL, were selected to conduct this study. The ethics committee of the Institute had approved the project and informed consent was taken from all subjects included in the study. Total population covered for initial screening was 9445 which were clinically examined for fever, present and past history of kala-azar, weight loss, splenomegaly and hepatomegaly.

### Inclusion criteria

Participants of either sex aged more than or equal to (≥) 6 years with or without family history of VL were included from the sampled villages.

### Exclusion criteria

Exclusion criteria were haemoglobin(Hb)<5 g/100 mL; white blood count <1,000/mm^3;^ thrombocyte count <50,000; alanine transaminase, aspirate transaminase, and alkaline phosphatase ≥2 times upper limit of normal, serum creatinine or blood urea nitrogen ≥1.5 times upper limit of normal. Patient’ssero-positive for HIV, hepatitis B and C, tuberculosis (TB), malaria, kidney disease, hypersensitivity to amphotericin B or inactive ingredients of the amphotericin B formulation were also excluded.The rK39-negative patients were classified as non-kala-azar cases and managed by the testing physician for an alternative illness.

### Experimental design

Following preliminary clinical and haematological investigations all were screened by rK39, immuno-chromatographic test (ICT) and the asymptomatic cases who defined an endemic areas individual with rK39 positive without signs and symptoms were segregated under threecategories of anti-leishmanial antibody titre category 1 (<1:800), category 2 (1:1600–1:6400) and category 3 (>1:6400). These categories were based on DAT result.Among the subjects category 1 and category 2, DAT positive sero-positives underwent confirmation of VL by qPCR and immunological tests (ADA level, IFN-γ, IL-10 and TNF-α levels). The subjects were followed up clinically, parasitologically (qPCR) and immunologically (Adenosine deaminase Activity, IFN-γ, IL-10 and TNF-α) at 6- month follow up.

For all these investigations initially, one spot of blood from all selected individuals including the healthy controls the treated and untreated VL patients were immediately tested, using an rk-39 strip. Further, samples of figure prick blood from these subjects was sent at immunology division of Rajendra Memorial Research Institute of Medical Sciences (ICMR-RMRIMS), Patna for assessment of the DAT titre. After obtaining informed consent, 5ml whole blood was also collected by venepuncture in a plain and sodium-EDTA vacutainer (BD Biosciences, USA) from each study subject. Serum was extracted for both rK-39 and the DAT while plasma was extracted from sodium-EDTA vacutainer for quantitative assessment of cytokine through sandwich ELISA.

### rK39 strip test

Kala-azar detect, a commercial version of the immuno-chromatographic strip test based on the rK39 antigen (In Bios International, Seattle, WA) was initially applied for screening all 5794 subjects. Briefly, the test was performed on a drop of blood, placed on the absorbent pad on nitrocellulose membrane, followed by addition of two drops of chase buffer. Results were considered positive when, two pink lines(one control and one test) appeared after 10 minutes.

### DAT

The antigen for the DAT was prepared and test performed as previously reported [23, 34]. For this, around 5 mm punched filter with dried blood spot(whatman filter paper4) was kept overnight in DAT diluent for elution. A sample was considered positive if it had a titre ≥ 1:800[4].

### Determination of ADA level

Determined the ADA activity by ADA-MTP kit (Tulip Diagnostic India) according to the instructions illustrated in manufacturer protocol. In brief, fresh blood samples weretaken out in sodium- EDTA vacutainer (BD Bioscience) which wascentrifuged at 3000rpm for 2 min at 4°C. The upper plasma was then transferred to a sterile cryo vial, flash froze in liquid nitrogen and stored at -80°C till further use. The fluorescent intensity was determined at Ex/Bn 535/587 by carry Eclipse Fluorescance Spectrophotometer (Agilent technologies, CA, USA). The same plasma protein concentration was used in each assay.

### ELISA analysis for different cytokine (IFN-γ, IL-10& TNF-α)

The total level of cytokines(IFN-γ, IL-10& TNF-α) was measured by BD OptiEIA (BD Bioscience SD, USA) ELISA kits using the culture supernatants of antigen stimulated PBMC culture and post setup of culture according to instructions provided by the manufacturer. The colour intensity was measured at 450nm by iMARK Microplate Reader (Biorad).Cells (PBMCs) were obtained following Histopaque 1077(Sigma Helrich, Dorset, UK) density gradient centrifugation at 400g for 30 min at 20˚C from the heparinised peripheral blood of the subjects. Following washing twice in RPMI 1640, PBMCs were suspended in complete RPMI medium at a concentration of 1×10^6^ cells/ml. These cells (1×10^6^ cells/ml) were cultured at a concentration of 2×10^5^ cells/ml in 24-well tissue culture plates in complete medium for 16h at 37˚C and 5% Co_2_. Cell culture supernatants were analysedfor cytokines by ELISA techniques according to the manufacturer’s instruction [35]. The detection limits were 5.6pg/ml for IFN-γ(EMD Milipore, Billercia, MA), 2pg/ml for IL-10(EMD Milipore) and 3.5pg/ml for TNF-α(EMD Milipore). All samples were simultaneously run in triplicate cells. Cytokine absorbence was read at a wavelength of 450nm in an enzyme-linked immunosorbent assay(ELISA) reader (Bio-Rad, iMark, Gurgaon, India).

### Quantitation of parasites in asymptomatic patients(qPCR)

DNA was isolated from blood using a QIAamp DNA blood mini kit (Qiagen, Hilden, Germany) as per the manufacturer’s instructions. Blood sample were kept in ATL buffer and protease K and incubated at 56°C for 2 hours before further processing according to the manufacturer’s instructions. Extracted DNA samples were stored at –20°C until real-time PCR.To determine the parasitic load, we performed the Real Time PCR (Roche) using SYBR green (Roche) chemistry by (kDNA) using a kDNA gene-specific primer (F-5’-TCTGTGGCCCATTTGTTGTA-3’,andR-5’-CATTTTTGGGTTTTCGGAGA-3’).To quantify the parasitic load of each sample was extrapolated to standard curve using 10 fold serially diluted *L*. *donovani* parasites DNA corresponding to 10^4^ to 0.1parasites per reaction as shown in our previous paper [36–39].The cycling conditions were as follows: 1 cycle at 95°C for 3 min and 40 cycles of 95°C for 15 s (denaturation), 58°C for 30 s (annealing), and 72°C for 30 s (extension). The fluorescence signal was captured at the end of each cycle using the SYBR channel (490-nm wavelength for excitation and 525-nm wavelength for emission).The threshold cycle value (Ct) was calculated for each sample by determining the point at which fluorescence exceed the thresholds limit and calculated the parasites/μg DNA.

### Data management, quality assurance and statistical analysis

The entire questionnaire was scrutinized for accuracy and consistency. Frequency and distribution checks were applied to access the range of values and identify missing data. Checks were also applied during designing the Data Entry Program to reduce the data inaccuracy. All forms of suspected cases were double-entered and errors validated against original record. In addition, RMRI field team was looked into the entire data collection process using independent vehicles, and was conducted systematic back checks. Finally, the medical teams visit clinically and haematologically suspected subjects and reconfirmed that they do indeed meet the definition of suspected cases. All the structured/semi-structured questionnaires used under this survey were pre-tested. After verification, each record was given to data entry operators for data entry. Data were entered, cleaned using Epi Info Version 3.3.2 (CDC Atlanta).

Data was extracted from the structured/semi-structured questionnaires using a structured data sheet specially designed for the purpose. Data extraction was conducted by the project workers, working for the project. Before embarking on the data collection process, field workers attended a two-days training provided by the principal investigator on how to fill the structured/semi-structured questionnaires. To ensure data quality, the following measures were taken: (a) additional two-days training was given to field supervisor before the start of data extraction, (b) the overall activities of data extraction was monitored by the principal investigator, and there was strict supervision during data collection also, (c) all completed datasets were examined by the principal investigator for completeness, and (d) from the data extracted from each structured/semi-structured questionnaires, 5% of the sample was randomly selected and validated against the registers/records by the principal investigator. The extracted data was verified from two sources i.e. from door-to-door visit of clinically and haematologically suspected subjects.

Data were analyzed using Graph Pad Prism 5 (USA software) and presented as Mean ± SD. Student’s two-tailed paired t-test or unpaired t test was used to determine the difference between the groups studied. P value <0.05 were considered statistically significant for all the analysis.

## Results

### Baseline field survey

Based on health monitoring government data, it was assumed that about 10,000 population was required to be surveyed to get sizeable number of asymptomatic cases from highly VL affected district of Bihar, India. As such, one highly endemic village was identified within the identified PHC i.e.Garkha and Dariapur. House to house survey was conducted to collect demographic data through pretested questionnaires. Proper informed consent was obtained from head / representative of the family.

Altogether 5794 individuals who were aged ≥6 years in the selected villages were screened with rk-39 strip in order to identify the baseline asymptomatic carriers of *L*. *donovani* infection. Out of that, 2835 (49%) was male and 2959 (51%) was females. Among them, maximum (38%) belonged to 6–15 years of age, followed by 25% in the age group of 16–30 years. The minimum and maximum age of the participant was 6 and 72 years respectively with mean age of 37 years. A total of 308 individuals were found to be rk-39 sero-positive which comprised 188 individuals having past history of kala-azar. Remaining 120 sera samples from a total of 308 rk-39 positive individuals had no previous history of kala-azar.

### Detection of sub-clinical infection with *L*. *donovani*

**Fig 1:**
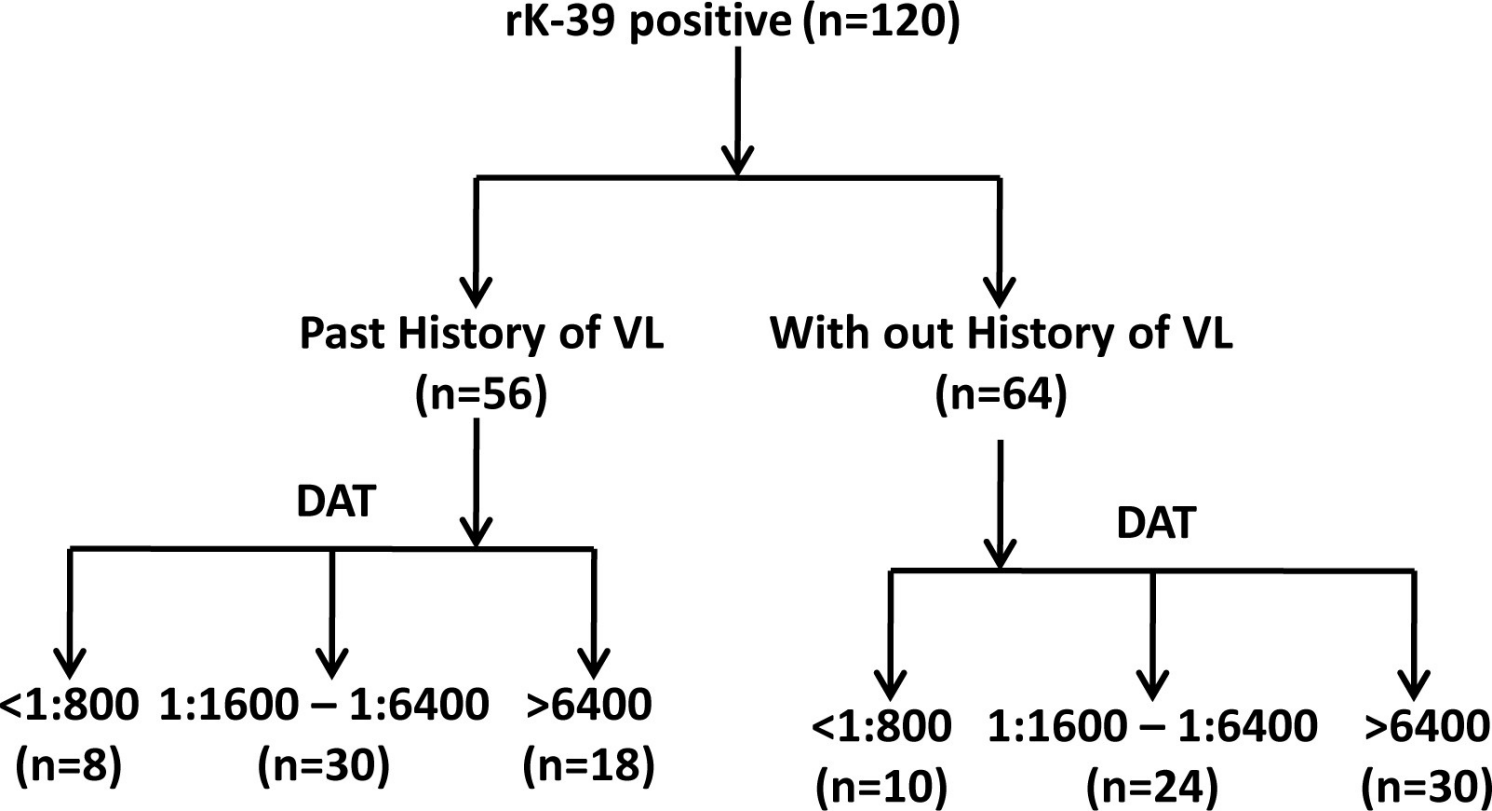
Flow diagram of serological results.

Based on the anti-leishmania antibody titres observed in the DAT, those 308 rk-39 sero-positive subjects were divided into three groups; those that were DAT negatives (<1:800) (DAT _neg_), a positive DAT with a low titre range (1: 1600–1: 6400) (DAT _pos_Low) and a positive DAT (>1:6400; DAT_pos_High) with maximum titre range. Based on such categorization, we identified 24 asymptomatic suspects under the DAT _Pos_ Low / titre range, 30 asymptomatic subjects under the DAT _Pos_High titre range and 10 suspects under DAT _Neg_ titre range. Fifty six (56) individuals, showing rk-39 positivity out of 308 suspects were having previous history of kala-azar, comprising (N = 30) DAT _Pos_Low titre range and (N = 18) DAT _pos_ High titre range. Comparison of parasite load byqPCR with 56 individuals with previous history of Kala-azar revealed that out of 56 such individuals, 15 in DAT_Pos_Low and 8 in DAT_Pos_ High were qPCRpositive.We found direct correlation for increased DAT titre range with *Ld*/μg DNA in qPCR. For instance, where rK-39 positive suspects were in DAT _Pos_Lowtitre, the average *Ld*/μg DNA was >94 whereas, when it increased (DAT>1:6400), the average *Ld* DNA/μg became ≥232 *Ld*/μg DNA. Similarly, in individuals with no previous history of VL, out of 64 subjects, we observed 6 qPCR positive individuals in DAT _Pos_Lowand 13 qPCR positive in DAT _Pos_High. Based on such categorization, we could finally identify 42 asymptomatic suspects (Table 1, Fig 1).

**Table 1:** Development of endemic healthy control into symptomatic VL at the end of 6 month follow up (rK39, DAT and qPCR analysis).

Subjects	rK39 positive suspects (120)				Asymptomatic to VL conversion (6 months)
	DAT	Number	qPCR negative (Ld/μg DNA)	qPCR Positive (Ld/μgDNA)	
	Anti- Leishmania titre (Baseline)				
Past history of VL (n = 56)	<1:800	8	-	-	-
	1:1600–1:6400	30	15	15	3
	>1:6400	18	10	8	4
				
No history of VL (n = 64)	<1:800	10	-	-	-
	1:1600–1:6400	24	18	6	
	>1:6400	30	17	13	3
				
Confirmed VL (n = 50) Control	<1:800	-	-	-	NA
	1:1600–1:6400	30	-	30	
	>1:6400	20	-	20	

• <40, Ld/μg DNA was considered negative samples.

• Only qPCR positive individuals were considered for further studies.

### Monitoring of serum ADA level in VL patients, endemic asymptomatic subjects and in endemic asymptomatic subjects converted into VL at 6 months follow-up

**Fig 2:**
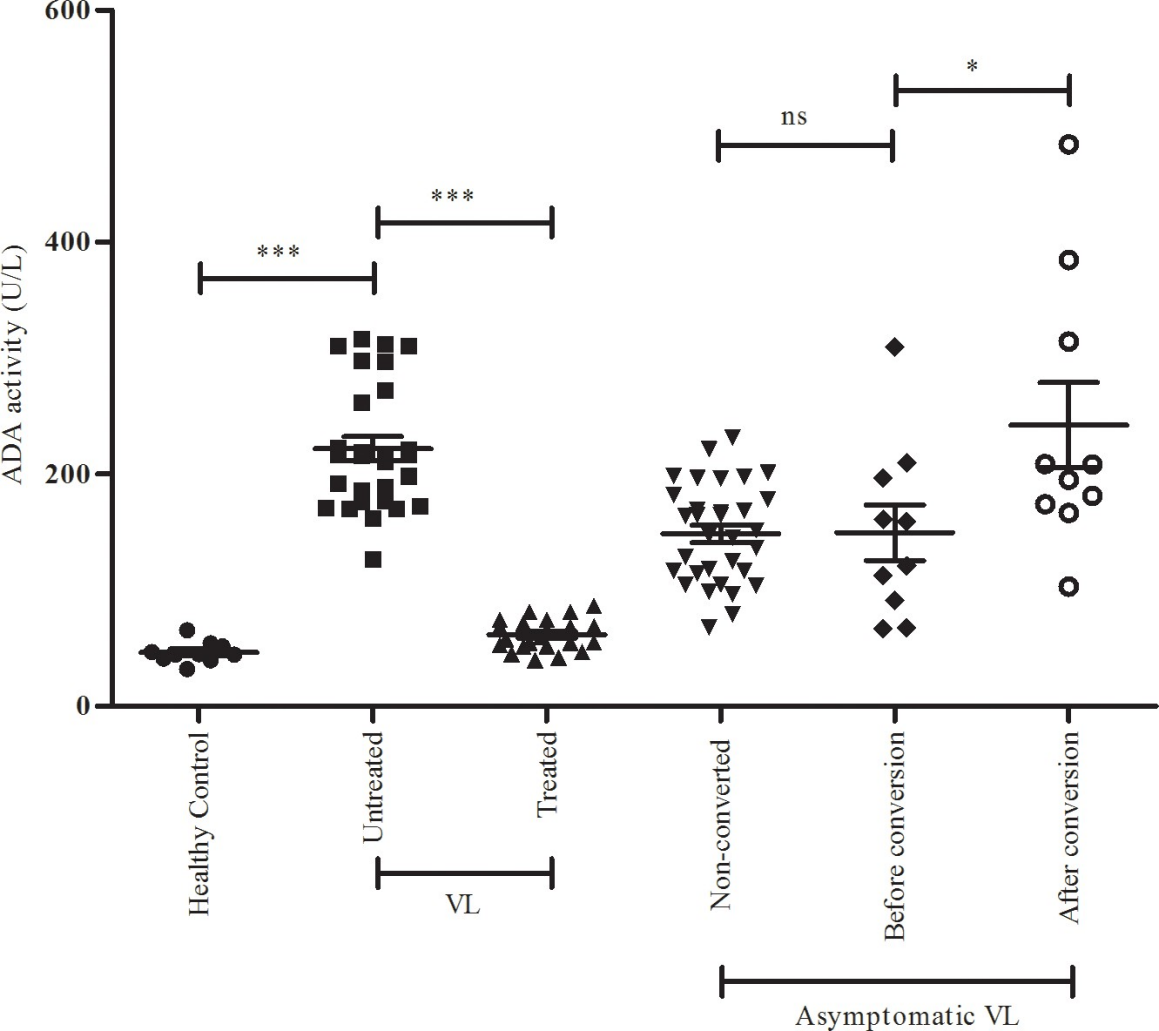
Serum ADA activity of healthy subject (n = 10), VL untreated (n = 28), VL treated (n = 23), Non-converted (n = 32) asymptomatic VL and after conversion into VL (n = 10) (Before conversion and after conversion (6 month)). Non-significant (ns), ***P<0.0001, *P<0.05.

In a supplementary experiment it was shown that serum ADA activity in VL patients at before treatment remained significantly high compared to after treated and healthy subjects (Table 2, Fig 2 p<0.0001).

**Table 2:** Serum ADA activity of active VL cases before and after treatment (Serum ADA activity (A) of healthy subject (n = 10), VL untreated (n = 28) and VL treated (n = 23)).

Subject	Number	ADA activity(U/L)	P value	
Healthy control	10	46.54±9.07	<0.0001	
VL untreated	28	222.10±54.29		<0.0001
VL treated	23	61.77±13.23		

Serum ADA activity in endemic healthy individuals was observed low and in normal range. It has also previously been shown that ADA is secreted against pathogens [28] and it also observed elevated during VL [1], thereby, we anticipated that fluctuated and altered ADA activity should possibly reflect the presence of parasite in a few asymptomatic cases, being converted into VL. As such, we monitored serum ADA activity in endemic symptomatic subjects before and after conversion (Table 3, Fig 2). As discussed in the preceding section,we had identified a total of 10 rK-39 suspects who had shown high anti-Leishmania DAT titre, were also qPCR positive. Assuming those 10 suspects as asymptomatic cases of VL from endemic area of VL in Bihar state, India, we next tried to know the status of ADA activity in such endemic asymptomatic subjects (Table 3, Fig 2). The data revealed that all 10 qPCR positive asymptomatic subjects were found to possess elevated serumADA level (Table 3, Fig 2 p = 0.0284).

**Table 3:** Serum ADA activity in asymptomatic VL and after conversion into VL (Serum ADA activity of asymptomatic VL (n = 10) and after conversion into active VL (n = 10)).

Asymptomatic VL (rK39+, DAT+, qPCR+) (n = 42)		ADA activity(U/L)	P value	
Non-converted (n = 32)		148.90 ± 42.47	0.9642	
Converted into VL (n = 10)	Before conversion	149.70 ± 75.26		0.0284
	After conversion (6 months)	242.30 ± 116.40		

• P-value 0.9642 corresponds to before conversion set of data.

### Serum ADA level in VL patients in their pre and post treatment stage and endemic asymptomatic subjects

VL patient group produced significantly higher level of serum ADA at pre-treatment stage than was observed to the corresponding value of ADA in their respective post treatment stage and healthy control (Table 2, p<0.0001). Anticipating that some of the asymptomatic subjects might lead to clinical manifestation of VL with increasing time period, we examined serum ADA activity of asymptomatic individuals. Results obtained were interesting and ADA activity observed was not consistent, between the initial survey and after 6-month follow-up, ten (10) subjects (All of whom were subsequently found p-PCR positive and sero-positives) developed VL manifestation such as irregular fever, malaise, loss of weight and splenomegaly. The RMRIMS’s clinicianshadparasitologically confirmed these individuals asVL. Sera samples of such cases was found to produce very high ADA level to their respective baseline ADA activity (p = 0.0284). The remaining 32 asymptomatic subjects who were found qPCR positive at baseline did not complain of fever or any other symptom during the study period.

### Cytokine trend in asymptomatic VL and during conversion from asymptomatic to symptomatic VL with appearance of clinical manifestations of visceral leishmaniasis

**Fig 3:**
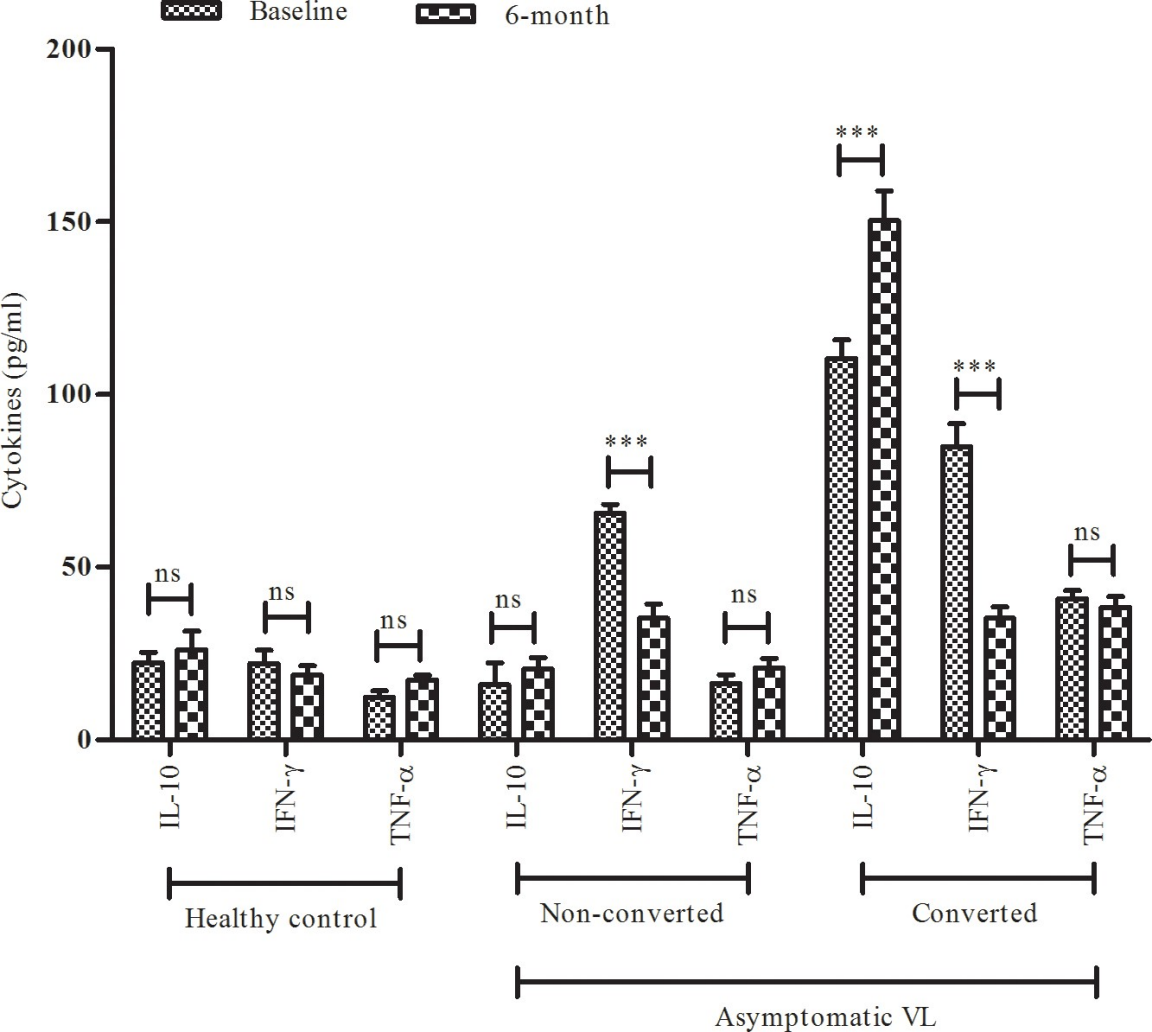
The relationship between cytokine production (IL-10, IFN-γ and TNF-α) and positivity in RT-PCR indicating subclinical infection and during the development of symptomatic visceral leishmaniasis (VL) in 10 asymptomatic residents of an endemic area of Bihar. Healthy subject (n = 10), asymptomatic VL (non-converted (n = 32) and converted into VL (n = 10)).Data were represented as mean ± SD, P≤0.05 were considered as significant.*P≤0.05, **P≤0.01, ***P ≤ 0.001, ns (non-significant).

As illustrated in Fig 3, control group produced relatively low IL-10, IFN-γ and TNF-α. Endemic asymptomatic patients produced significantly higher quantitative yield of IL-10, while IL-10 produced by non-converted asymptomatic suspects (DAT, rK39 positive and RT-PCR positive n = 32) was significantly low. As soon as, such asymptomatic patients developed full clinical manifestations of VL(6 months), IL-10 produced by these patients once again increased and became 1.36 fold higher compared to quantity of IL-10 produced by asymptomatic suspects, not converted into VL, and in them, IL-10 was over all 7.31 fold down.

On the other hand, both categories of asymptomatic VL (converted verses non-converted) individuals produced significantly higher IFN-γ then were observed in healthy subjects. Comparatively, the IFN-γ levels in asymptomatic VL patients were higher at base-line then in non-converted asymptomatic suspects. The scenario became reverse and unlike to IL-10, there was observed decrease of about 2.40 fold in total yield of IFN-γ, when converted patients follow-up at 6 months on appearance of full blown clinical manifestations. The mean level of IFN-γ in non-converted endemic asymptomatic individuals was at base-line and at 6 month follow-up remained low and comparable.

Asymptomatic VL patients consistently were observed to produce higher yield of TNF-α before and after conversion. On the other hand, asymptomatic individuals who failed to convert into clinical VL were observed with low yield of TNF-α which however was statistically non-significant.

## Discussion

We identified potential biomarkers in VL patients, whose levels may be correlated with the conversion of asymptomatic patients to become symptomatic up to clinical manifestation and disease. Advancement towards the disease is likely due to increased *L*.*donovani*, which may modulate host CMI response. Many VL patients become immuno-compromised and die due to secondary infection. After the treatment failure somehow facilitates the problem as early in asymptomatic cases not identified. We assessed the pattern of cellure immune response in the community in VL endemic areas to predict the presence of sub-clinical infection [5]. Cytokines that regulate cellular immunity are the critical checkpoints to determine resistance or susceptibility to disease [40, 41]. Amongst cytokines IFN- γ has a pivoted role in protection in all forms of leishmaniasis [29, 42]. This cytokine works in tandem with TNF-α to exert leishmanicidal activitythrough reactive oxygen species (ROS) and reactive nitrogen species (RNS) generation are available [8, 43]. VL patients, however, release inadequate IFN-γ and develop various clinical conditions such as splenomegaly pancytopenia, anemia and disseminated hemorrhages [8, 31, 44–46] in the presence of excess IL-10 production [25, 32, 47, 48]. Adenosine produced by macrophages has a critical role in IL-10 production during *L*.*donovani* infection [49]. Adenosine was reported to suppress the release of IL-12 and nitric oxide production during infection [28,50]. Therefore, we measured serum ADA activity and cytokine profile (IL-10, TNF-α, and IFN- γ) at baseline and at 6-month follow-up in a community.

Direct diagnostic approaches for parasite detection in either spleen or bone marrow smears are usually not preferred for community screening due to invasiveness and ethnic consideration, hence diagnosis gets delayed. Antibody detection kits are usually used (rK39, rk26) for sero- surveillance [4, 23]. We identified 308 individuals out of 5794 screened population as rK39 strip test positive. Specifically, the rK39 strip test is commonly used worldwide, despite its low sensitivity in Africa [51]. More over sensitivity of rK39 in Sudan is only 67% [52]. Therefore, we also further validated such sero-positives through direct agglutination test (DAT). We performed DAT as the next step detection method for several reasons. This test is an easy serological method and even less skilled health workers can perform it at primary health care level [19, 53, 54]. More importantly, the inclusion of 2- Mercaptoethanol in the diluents makes DAT more compatible to identify *L*.*donovani* specific IgM antibodies which get expressed at the earliest, soon after individuals get exposed to the parasite [55, 56]. Our concern in this study was straight forward to detect and filter asymptomatic individuals with VL from a large number of the population residing in the VL endemic area. Before applying DAT for community screening, we had performed a Chequer board analysis, where an anti- leishmania titer of 1:800 was observed optimal as the cut-off with 91.7% sensitivity and 100% specificity for specific diagnosis of VL [4]. DAT analysis gave a validated proof that only 120 individuals out of 308 rK39sero-positives were exposed in the real sense, in whom DAT become able to identify parasite-specific antibodies. The few false positives recorded by rK39 amongst the community can be due to advanced or very early infection. While the strip test appears less specific than the DAT, it is recommended to use the combined application of rK39 and DAT for optimizing identification of very early (asymptomatic) infection of VL. Whether or not, these 120 sero-positives (rK39, DAT), actually represented asymptomatic VL?. This question arises due to certain drawbacks of antibody-based diagnostic tools which sometimes become positive in recovered VL subjects at different time span after cure.

In subsequent experiments, we went on to perform quantitative PCR on serologically filtered 120 DAT positive asymptomatic VL subjects. In similar to our findings, some previous studies also suggested that transmission of *L*. *donovani* in Bihar is focal (120 of the 308 rK39 positive, who lived in the endemic region of Bihar); 56 lived in households with a family history of VL and 64 lived in households with no history of VL. Anyway, both groups (those with history of VL and those with no history of VL), living in the same endemic region, were generally at high risk of infection.

Forty-two (42) seropositive suspects out of 102 DAT positive showed qPCR positivity and all had recorded more than 40 Ld/mg/DNA. From qPCR analysis, unlike sero-positives, we get evidence for the first time in this study that suspected individuals living in households with family history, were at particularly high risk of infection. The usefulness of quantitative PCR in epidemiology has recently been shown for the early detection of visceral leishmaniasis cases [11, 12, 57, 58]. This analysis helped us to identify 42 asymptomatic VL subjects and it necessitated to explore, how many of them converted into disease as well as the factors that promoted conversion of asymptomatic individuals to VL cases. The follow-up analysis revealed that most (32 of the 42 qPCR positives) cleared their infection within 6 months. Many asymptomatic subjects become sero-negative, without developing VL, within 12–36 months [5]. It has been shown earlier that some of them showing serological reactivity to *Leishmania* possess strong cellular immunity, and this helps to clear parasite from their blood before inducing disease in such cases [59]. Curiously, among the 42 qPCR positive subjects also found sero-positive at the baseline survey, only 7(seven) of the 23 from households with a family history of VL and 3 of the 19 from households with no family history of VL developed the symptomatic disease at 6 months.

Therefore altogether 10 of 42 (23.80%) asymptomatic cases having rk-39, DAT and qPCR positivity converted into disease during this study project. This approach appears to be more authentic because if the rk-39 strip test and DAT would be considered, individually as sole criteria for baseline survey, chances of detecting difficult cases with parasitemia in resource-poor rural areas become low. As a result, only 3.27% (10 of 308) and 8.33% (10 of 120), considered positive at baseline converted from asymptomatic to symptomatic VL. The study period remained during the period of VL transmission (February- November) and it is anticipated that most *L*.*donovani* infections, which convert to disease, take a time of 6 months in the endemic areas of Bihar [4]. We surmise from our data that qPCR done on samples, filtered initially based on rk-39 and DAT can be useful in the detection of early parasitemia and conversion to symptomatic VL.

In our study those 10 patients who converted into VL among them the minimum Ct value is ~32. Asides host, parasites were also modulating the host cellular immune response during conversion from early parasitemia in asymptomatic VL patients to established parasitemia in grown-up VL patients with various clinical manifestations. Several reports have also suggested that parasitic micro-organisms seek the help of adenosine and adenosine receptors during the internalization of parasite and to skip the protective immune response of the host [60, 61]. It would be interesting to add here that there are two types of ADA activities that operate under the influence of iso-enzymes in the host during an inflammatory response. One iso-enzyme, ADA-1, is responsible for protective immune response and is present in all tissues. In contrast to it, we reported the presence of A2B receptors in monocytes and macrophages on VL patients and the majority of such A2B+ expressing monocytes were in general high IL-10 producers [62]. The course of ADA activities observed in VL patients is due to the activation of other principal iso-enzymes, ADA-2. ADA-2 is only present on monocytes and macrophages and its activation is triggered in response to the pathogen [28, 63].

The serum adenosine deaminase (ADA) activity was therefore assessed as this can potentially influence host suppressive Th2 immune response (IL-10) during the progression of early parasitemia in asymptomatic individuals. Initial testing was done at RMRIMS, Patna in VL patients before and after treatment. The findings reflected more ADA activity in active and untreated VL patients compared to in VL patients after treatment and control. Asymptomatic patients identified at baseline screening were observed with consistently high serum ADA activity. The rest of the individuals had their ADA level to be par with the normal range as observed in control. ADA activities remained high in both categories of DAT and rK39 positive subjects are they were qPCR positive or negative. Therefore even at the onset of the immune response, when individuals get exposed demonstrate threshold antibodies to *Leishmania* antigen after biting of infected sandfly and in them, ADA level gets increased. Earlier also, it has been reported that serum ADA activities can be used as a marker to monitor the progression of asymptomatic to symptomatic VL.

We reported earlier the necessity of A2B adenosine receptors in the severity of VL infection [62]. Evidence was also drawn during this study that when monocytes were infected in the presence of adenosine, the rate of *L*. *donovani* infection increased, whereas *Leishmania* infection became abrogated after blocking the adenosine receptor [62]. Our result corroborates the previous finding in context to the precise link between load and ADA levels. More interestingly, the production of IL-10 remained significantly high in the group of asymptomatic patients, both before and after conversion than in the group of asymptomatic patients, who did not convert into VL. These findings seem to be very interesting when ADA activities in such patients are also considered.

Previous reports suggest that adenosine produced by ectonucleotidase, during activation of the ATP pathway, during infection contributes to the production of IL-10 and IL-6 [62].

We reported earlier the crucial role between IL-6 and up-regulated surface 3-ectonucleotidase activities synergizing with the anti inflammatory effect of IL-10 [48, 64] and STAT 3 phosphorylation [65]. Considering the growth and differentiation of inducing properties, IL-6 might also appear to correlate with increased ectonucleotidase activities in converted asymptomatic patients. We, however, did not examine the IL-6 level and concentrated on IL-10 but can hypothesize that IL-6 promotes the anti- inflammatory effect of IL-10 in leishmaniasis [51].The level of IFN-γ remained high, until the patients converted into symptomatic VL, and showed significantly low production. There was no change in the level of TNF-α during conversion into symptomatic patients.

Taken together above results suggest that combined application of rk-39, DAT and R T–PCR can help detect asymptomatic VL patients and also that asymptomatic VL patients have a big role in the VL transmission cycle in resource-poor rural areas of Bihar. Targeting adenosine and detecting ADA level can help us to monitor the progression of patients from early parasitemia in asymptomatic VL to clinical disease, since IL-10 was observed in high concentration, during conversion into clinical disease; IL-10 level concurrent to serum ADA activity can help to facilitate early intervention and interruption of disease transmission. Although the cohort tested in our study did not include population-specific genotype markers but the inclusion of such parameters could indeed foster prediction by combining molecular diagnostics with serodiagnostic. In one of the recent most studies on asymptomatic VL, were able to link several HLA-DRβ allele groups with the progression of VL [66].

### Ethics statement

This study was approved by the Institutional Ethics Committee of RMRIMS, Patna vide approval No.01/IEC/2014 dated 28^th^January, 2014.Participants of either sex aged more than or equal to (≥) 6 years with or without family history of VL were included in this study. The study specific written informed consent was obtained from the study participant. In case of minor (>6 years and <18 years), written informed consent was obtained from their parents/ guardians.
